# Sleep-disordered breathing in patients with heart failure with preserved left ventricular ejection fraction

**DOI:** 10.1007/s11325-026-03635-w

**Published:** 2026-03-13

**Authors:** Cornelia Grimm, Christian Viniol, Mikail Degerli, Olaf Hildebrandt, Karl Kesper, Ulrich Koehler, Wolfram Grimm, Mariana Parahuleva

**Affiliations:** 1 Department of Cardiology, University Hospital of Marburg and Gießen, Philipps-University Marburg, Marburg, Germany; 2 Sleep Disorder Unit of the Department of Pneumology, University Hospital of Marburg and Gießen, Philipps-University Marburg, Marburg, Germany

**Keywords:** Sleep-disordered breathing, Heart failure, Preserved left ventricular ejection fraction, Obstructive sleep apnea, Central sleep apnea

## Abstract

**Purpose:**

Data on sleep-disordered breathing (SDB) in patients with heart failure with preserved left ventricular ejection fraction (HFpEF) are sparse. Therefore, we aimed to determine the prevalence and characteristics of SDB in a large patient cohort with HFpEF.

**Methods:**

A total of 233 patients with HFpEF were prospectively enrolled at our cardiology outpatient department after excluding patients with a history of SDB. The presence of moderate to severe SDB with an apnea-hypopnea index ≥ 15/h was determined using cardiorespiratory polygraphy. All patients underwent assessment of HFpEF comorbidities, comprehensive echocardiographic studies, NT-proBNP levels and calculation of HFpEF-scores.

**Results:**

SDB was found in 97 of 233 patients (42%) with predominantly obstructive sleep apnea (OSA) in 64 patients (27%) and predominantly central sleep apnea (CSA) in 33 patients (14%). Male sex, body mass index, NYHA heart failure class III, NT-proBNP levels, HFpEF scores, chronic kidney disease, coronary artery disease, left ventricular (LV) mass index and E/E’ ratio by echocardiography were significant predictors of SDB by univariate analysis. Patients with predominantly CSA were significantly older and had higher NT-proBNP levels and a higher NYHA heart failure class than patients with OSA. Patients with OSA had a significantly higher body mass index (BMI) and a higher Epworth Sleepiness score. Multivariate analysis revealed male gender, BMI and LV mass index as significant predictors for OSA, whereas CSA was associated with a higher HFA-PEFF score and male gender.

**Conclusions:**

Moderate to severe SDB is a frequent comorbidity in patients with HFpEF. Predominantly OSA is more frequent than CSA with significant clinical differences between patients with OSA compared to CSA. Whereas OSA is associated with a higher BMI in addition to male gender, CSA is associated with more advanced heart failure as indicated by higher HFA-PEFF scores, NYHA heart failure class and NT-proBNP levels.

## Introduction

Sleep-disordered breathing (SDB) is an important underdiagnosed comorbidity in heart failure [1]. In contrast to heart failure with reduced left ventricular ejection fraction (HFrEF), only limited information is available on the prevalence of SDB in heart failure with preserved left ventricular ejection fraction (HFpEF) [2–12]. Furthermore, it is largely unknown which clinical characteristics distinguish patients with HFpEF and obstructive sleep apnea (OSA) or central sleep apnea (CSA) from patients with HFpEF without SDB. Therefore, we performed a prospective study in 233 patients who presented with HFpEF after patients with a history of SDB were excluded.

## Methods

### Patients

**Fig. 1:**
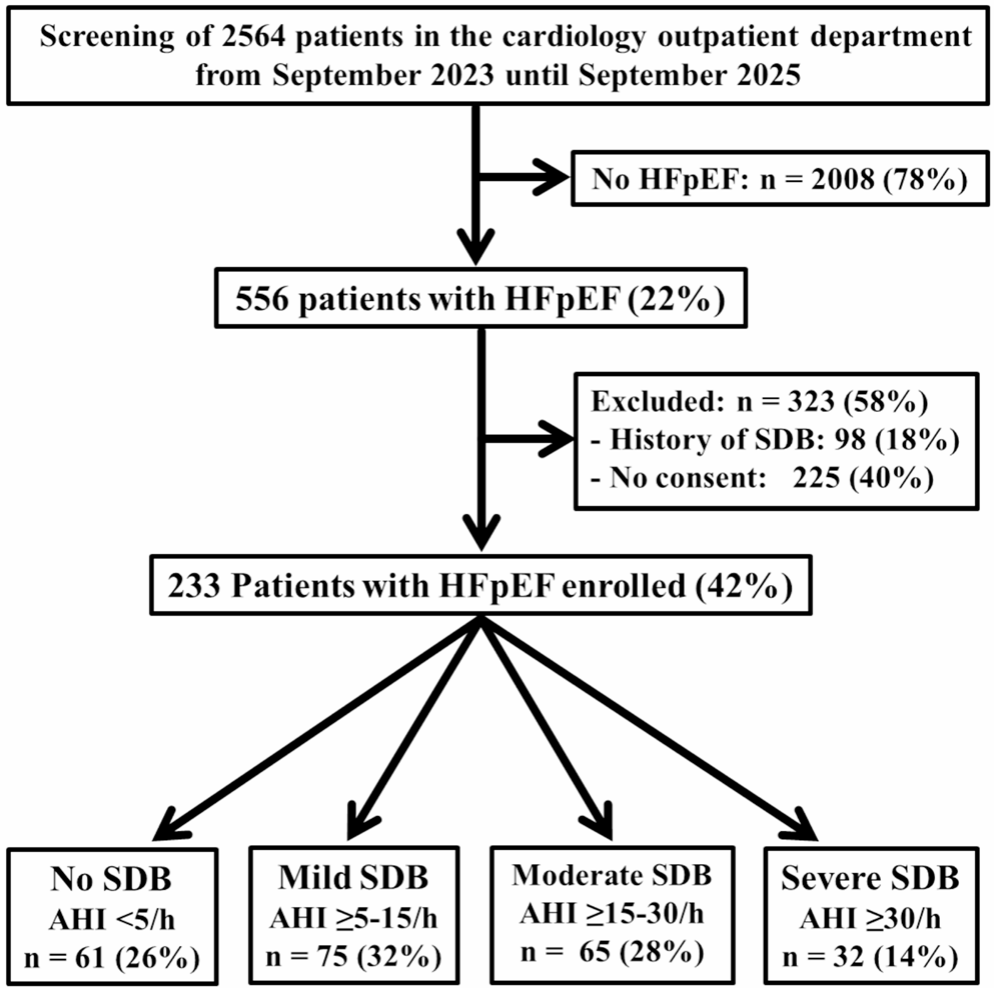
Study profile

From September 2023 until September 2025, a total of 233 adult patients with HFpEF confirmed according to current heart failure guideline criteria [13, 14] including extensive echocardiography and NT-proBNP determination in all patients were prospectively enrolled in this monocenter study at the cardiology outpatient department of our hospital after exclusion of 98 patients with a history of SDB diagnosed by a previous sleep study and exclusion of 225 patients who refused to give informed consent (Fig. 1). The study protocol was reviewed and approved by the ethics committee of the University of Marburg. Written informed consent was obtained from all study patients.

### Cardiorespiratory polygraphy

All study patients underwent screening of SDB by overnight polygraphy using the Samoa® device or the MiniScreen plus® or MinoScreen premium® devices, Löwenstein Medical, Bad Ems, Germany. All polygraphy devices used a nasal cannula to measure flow and snoring noises, a thermistor sensor for breathing, a finger sensor including a pulse oximeter for oxygen saturation and pulse frequency, and sensors for recording thorax movements in a chest belt and abdominal movements in an abdominal belt. Moderate to severe SDB was defined using an apnea-hypopnea index (AHI) ≥ 15/h. CSA was diagnosed when the number of central apneas exceeded the number of obstructive apneas in the presence of an AHI ≥ 15/h. Hypopnea was defined based on American Academy of Sleep Medicine criteria [15] as a ≥ 30–90% decrease in airflow versus baseline for ≥ 10 s with ≥ 4% oxygen desaturation. Apnea was defined as a ≥ 90% decrease in airflow for ≥ 10 s[15]. OSA was diagnosed in the presence of a predominantly obstructive rather than central apnea pattern.

All polygraphic recordings were scored by an experienced sleep technician or physician who was unaware of the patients’ clinical characteristics. The validated Epworth Sleepiness Scale (ESS) score was used to assess subjective daytime sleepiness. Patients were asked to rate the likelihood of falling asleep in eight common situations, resulting in a score from 0 as least sleepy to 24 as sleepiest. Excessive daytime sleepiness was defined using an ESS score ≥ 11[11, 16, 17].

### Echocardiography

Comprehensive two-dimensional echocardiographic examinations were performed according to the recommendations of the German Society of Cardiology [18] in all patients to determine LV ejection fraction, LV size, left atrial volume index, LV mass index, E/E’ ratio using Doppler echocardiography and tissue Doppler imaging, and estimation of systolic pulmonary artery pressure by measuring tricuspid regurgitation velocity. LV ejection fraction was measured in the apical four-chamber view and orthogonal two-chamber view using the disc summation method (modified Simpson’s rule algorithm). All echocardiographic studies were performed by cardiologists who were unaware of the sleep study results.

### Laboratory tests

N-terminal pro-brain natriuretic peptide (NT-proBNP) level testing as the most reliable biomarker for HFpEF diagnosis [13, 14, 19] was performed in all 233 patients at study enrollment. All blood samples were taken at study enrollment in the cardiology outpatient department. Blood samples were brought directly into our clinical chemistry laboratory for measurement of blood concentrations of NT-proBNP, creatinine, and several other routine laboratory parameters. Glomerular filtration rates were also determined and chronic kidney disease was diagnosed in the presence of at least two estimated glomerular filtration rates using the Modification of Diet in Renal Disease formula below 60 ml/min per 1.73 m^2^ with an interval of at least 3 months.

### H_2_FPEF- and HFA-PEFF scores

H_2_FPEF and HFA-PEFF scores were calculated in all patients. The H_2_FPEF score[20, 21], which ranges from 0 to 9 points, utilizes six clinical and echocardiographic characteristics including the comorbidities obesity, arterial hypertension and atrial fibrillation as previously described by Reddy et al.[19, 20] in detail. The H_2_FPEF score[20, 21] does not include NT-proBNP levels. The HFA-PEFF score[22], which ranges from 0 to 6 points, consists of a combination of detailed echocardiographic measurements and natriuretic peptide levels with prespecified cut-offs for NT-proBNP depending on the presence or absence of atrial fibrillation. Recommended echocardiographic criteria consist of functional markers including E/E’ ratio and estimated systolic pulmonary artery pressure based on tricuspid regurgitation velocity as well as morphological markers including left atrial size and LV mass index. In contrast to the H_2_FPEF score[20, 21], the HFA-PEFF score[22] is exclusively based on echocardiographic measurements and natriuretic peptide levels and does not include comorbidities like atrial fibrillation, obesity or arterial hypertension.

### Statistical analysis

Results are expressed as mean ± standard deviation or median values with interquartile range (IQR) for continuous variables as appropriate. Univariate comparisons of clinical characteristics, comorbidities, echocardiographic parameters and laboratory findings between patients with and without SDB and between patients with predominantly OSA versus CSA were performed using Student’s t-test or Mann-Whitney U test for continuous variables, and categorical values were compared using Chi-square tests or Fisher’s exact tests where appropriate. Multivariate logistic regression analysis including all clinical characteristics, comorbidities, echocardiographic parameters and laboratory findings listed in Table 1 was used to determine risk factors for predominantly OSA and CSA in HFpEF after adjustment for all patients’ medications listed in Table 1 as potential confounding factors. All probability values reported are two-sided, and a probability value of *P* < 0.05 was considered to indicate statistical significance. IBM^®^ SPSS-software version 29 was used for all statistical analyses.

**Table 1:** Clinical characteristics and comorbidities of 233 Patients with and without SDB

Characteristics	All Patients (*n* = 233)	AHI < 15/h (*n* = 136)	AHI ≥ 15/h (*n* = 97)	*P*-value
AHI, n/h	15.5 ± 14.3	6.5 ± 4.3	28.1 ± 13.9	< 0.01
ESS score	6.0 ± 3.5	5.1 ± 3.3	7.3 ± 3.3	< 0.01
ESS Score ≥ 11, n (%)	33 (14)	11 (8)	22 (23)	< 0.01
Age, years	67 ± 12	66 ± 12	68 ± 11	0.12
Men, n (%)	155 (67)	76 (56)	79 (81)	< 0.01
Body mass index, kg/m^2^	30.4 ± 6.3	29.7 ± 6.4	31.4 ± 6.0	< 0,05
NYHA class II (%)	144 (62)	91 (67)	53 (55)	0.04
NYHA class III (%)	89 (38)	45 (33)	44 (45)	
NT-proBNP, Median [IQR]	324 [169, 862]	286 [137, 605]	385 [234, 1005]	< 0,–01
**Comorbidities**,** n (%)**				
Arterial hypertension	210 (90)	118 (87)	92 (95)	0.07
Diabetes mellitus	77 (33)	35 (26)	42 (43)	< 0.01
High cholesterol	182 (78)	103 (76)	79 (81)	0.38
Chronic obstructive lung disease	59 (25)	33 (24)	26 (27)	0.77
History of smoking	108 (46)	57 (42)	51 (53)	0.14
History of stroke	14 (6)	9 (7)	5 (5)	0.78
Coronary artery disease	122 (52)	60 (44)	62 (64)	< 0.01
Aortic valve stenosis	28 (12)	12 (9)	16 (16)	0.08
Cardiac amyloidosis	6 (3)	3 (2)	3 (3)	0,69
Mitral regurgitation ≥2nd degree	23 (10)	10 (7)	13 (13)	0.18
Hypertrophic cardiomyopathy	6 (3)	5 (4)	1 (1)	0.41
Chronic kidney disease	83 (36)	41 (30)	42 (43)	0.04
History of atrial fibrillation	88 (38)	52 (38)	34 (35)	0.72
**Echocardiography**				
LV ejection fraction, %	59±7	59±7	58±7	0.34
LV end-diastolic diameter, mm	50 ± 5	49±5	50±4	0.11
LV mass index (g/m^2^)	139 ± 29	132 ± 27	148 ± 29	< 0.01
LA volume index (ml/m^2^)	41 ± 13	40 ± 14	42 ± 12	0.27
E/E’ ratio	14 ± 4	13 ± 4	15 ± 4	< 0.01
Systolic PA pressure (mmHg)	41 ± 7	40 ± 7	41 ± 7	0.13
**HFpEF-Scores**				
HFA-PEFF Score	5 [4, 6]	5 [3, 6]	6 [5, 7]	< 0,01
H_2_PEF Score	5 [4, 7]	5 [4, 7]	6 [5, 7]	< 0,01
**Medication**,** n (%)**				
ACE inhibitor, ARB or ARNI	204 (88)	115 (85)	89 (92)	0.12
Loop diuretics	65 (28)	32 (24)	33 (34)	0.08
Thiazides	81 (35)	44 (32)	37 (38)	0.44
sGLT2 inhibitors	104 (45)	52 (38)	52 (54)	0.03
Aldosterone antagonists	58 (25)	33 (24)	25 (26)	0.91
ß-Blockers	169 (73)	93 (68)	76 (78)	0.13
GLP1 agonists	12 (5)	6 (4)	6 (6)	0.56

AHI, apnea-hypopnea index; ESS, Epworth sleepiness score; GFR, glomerular filtration rate; IQR, interquartile range; NYHA, New York Heart Association;

## Results

### Clinical characteristics of 233 study patients with HFpEF

During a 24 months period, 233 study patients with HFpEF were included (Fig. 1). Of the 233 study patients, 155 patients (67%) were men and 78 (33%) were women (Table 1). The mean age was 67±12 years. The mean LV ejection fraction was 59±7%. The majority of patients were in NYHA class 2 (62%). The remaining patients were in NYHA class 3 (38%). Mean AHI in the total study population was 15.5 ± 14.3/h (median AHI: 12) and mean ESS score was 6.0 ± 3.5 with an excessive daytime sleepiness as indexed by an ESS score ≥ 11 was found in 33 patients (14%).

### Prevalence of SDB in 233 study patients with HFpEF

Moderate to severe SDB with an AHI ≥ 15/h was found in 97 of 233 patients (42%) (Table 1). Predominantly OSA was present in 64 of 233 patients (27%) and predominantly CSA was present in 33 of 233 patients (14%) (Table 2). Excessive daytime sleepiness with an ESS score ≥ 11 was found more frequently in patients with SDB compared to patients without SDB (23% versus 8%, p < 0.01). LV ejection fraction and LV size by echocardiography were similar in patients with and without SDB. Univariate comparison of patients with and without SDB revealed a significant association between SDB and male gender, body mass index, NYHA heart failure class, NT-proBNP levels, both HFpEF scores, chronic kidney disease and coronary artery disease as well as LV mass index and E/E’ ratio determined by echocardiography.

**Table 2:** Comparison of patients with CSA to patients with OSA

Characteristics	CSA (*n* = 33)	OSA (*n* = 64)	*P*-value
AHI, n/h	27.2 ± 11.6	28.6 ± 14.9	0.63
ESS score	6.2 ± 3.2	7.9 ± 3.4	0.02
Age, years	73 ± 10	66 ± 11	< 0.01
Men, n (%)	27 (82)	52 (81)	0.95
Body mass index, kg/m^2^	29.8 ± 4.7	32.3 ± 6.5	0.04
NYHA class II (%)	12 (36)	41 (64)	< 0.01
NYHA class III (%)	21 (64)	23 (36)	
NT-proBNP, Median [IQR]	641 [343, 1324]	308 [172, 887]	0.04
**Comorbidities**,** n (%)**			
Arterial hypertension	31 (94)	61 (95)	0.77
Diabetes mellitus	16 (48)	26 (41)	0.46
High cholesterol	26 (79)	53 (83)	0.63
Chronic obstructive lung disease	9 (27)	17 (27)	0.94
History of smoking	15 (45)	36 (56)	0.31
History of stroke	2 (6)	3 (9)	0.77
Coronary artery disease	24 (73)	37 (58)	0.15
Aortic valve stenosis	10 (30)	6 (9)	< 0.01
Cardiac amyloidosis	1 (3)	2 (3)	0.98
Mitral regurgitation ≥2nd degree	7 (21)	6 (9)	0.11
History of myocarditis	1 (3)	2 (3)	0.98
Hypertrophic cardiomyopathy	1 (3)	0 (0)	0.34
Chronic kidney disease	18 (55)	24 (38)	0.11
History of atrial fibrillation	14 (42)	20 (31)	0.27
**Echocardiography**			
LV ejection fraction, %	58±7	58±7	0.99
LV end-diastolic diameter, mm	50±4	50±4	0.98
LV mass index (g/m^2^)	147 ± 26	149 ± 31	0.81
LA volume index (ml/m^2^)	43 ± 13	41 ± 11	0.47
E/E’ ratio	15 ± 4	14 ± 4	0.46
Systolic PA pressure (mmHg)	43 ± 6	41 ± 7	0.19
**HFpEF-Scores**			
HFA-PEFF Score	5.6 ± 0.7	5.1 ± 0.9	< 0.01
H_2_PEF Score	6.2 ± 1.5	5.8 ± 1.7	0.19
**Medication**,** n (%)**			
ACE inhibitor, ARB or ARNI	31 (94)	58 (91)	0.57
Loop diuretics	16 (48)	17 (27)	0.03
Thiazides	12 (36)	25 (39)	0.79
sGLT2 inhibitors	21 (64)	31 (48)	0.16
Aldosterone antagonists	7 (21)	18 (28)	0.46
ß-Blockers	26 (79)	50 (78)	0.94
GLP1 agonists	1 (3)	5 (8)	0.35

AHI, apnea-hypopnea index; CSA, central sleep apnea; ESS, Epworth sleepiness score; GFR, glomerular filtration rate; IQR, interquartile range; LA, left atrium; LV, left ventricular; NYHA, New York Heart Association; OSA, obstructive sleep apnea; PA, pulmonary artery

### Comparison of patients with predominantly CSA versus predominantly OSA

**Fig. 2:**
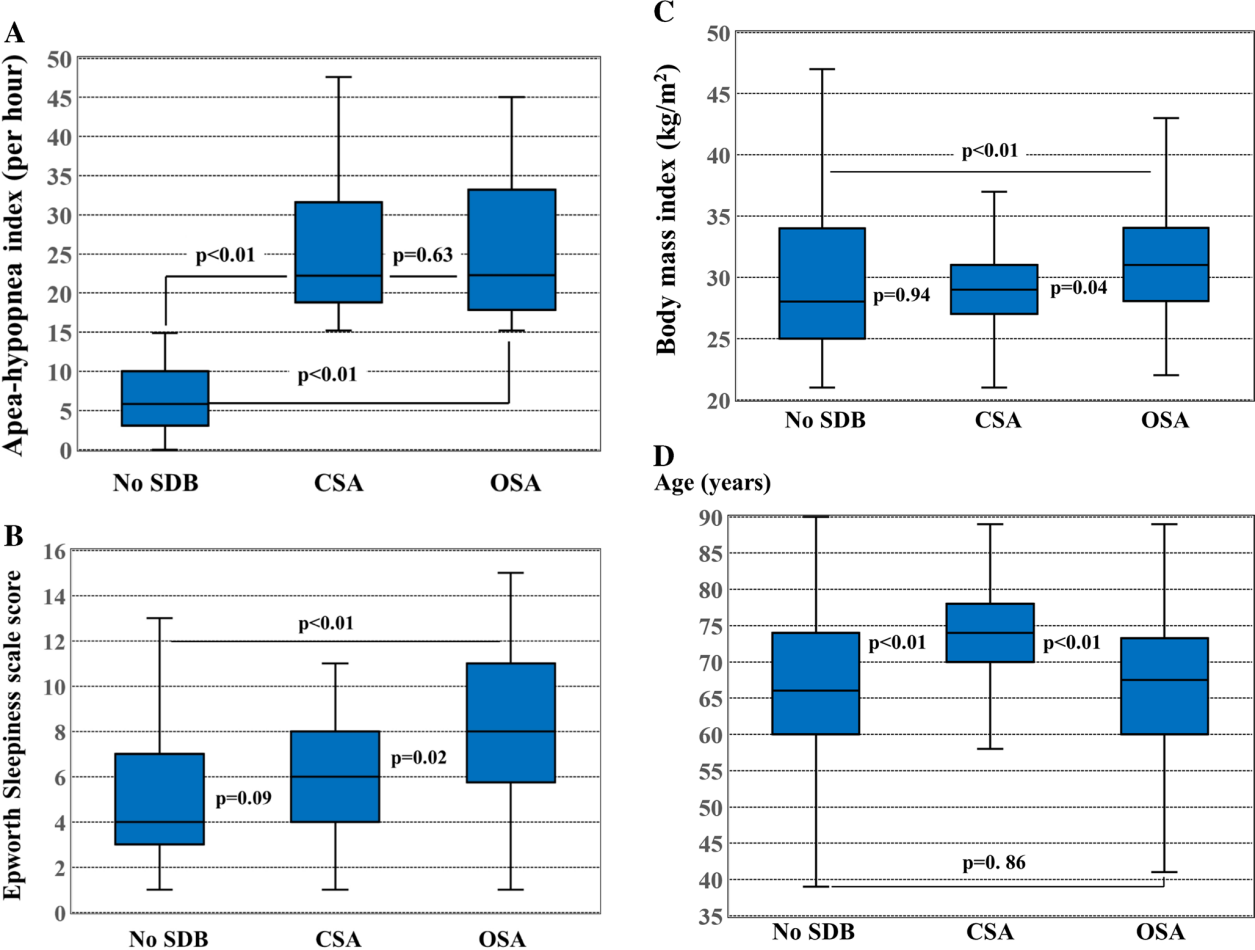
Box plots comparing patients without sleep-disordered breathing (SDB) to patients with central sleep apnea (CSA) and to patients with obstructive sleep apnea (OSA). (**A)** Apnea-hypopnea index was similar in CSA compared to OSA. (**B) **ESS score was significantly higher in OSA compared to CSA and no SDB. (**C**) BMI was significantly higher in OSA compared to CSA and to no SDB. (**D**) Patients with CSA were significantly older than patients with OSA or without SDB

**Fig. 3:**
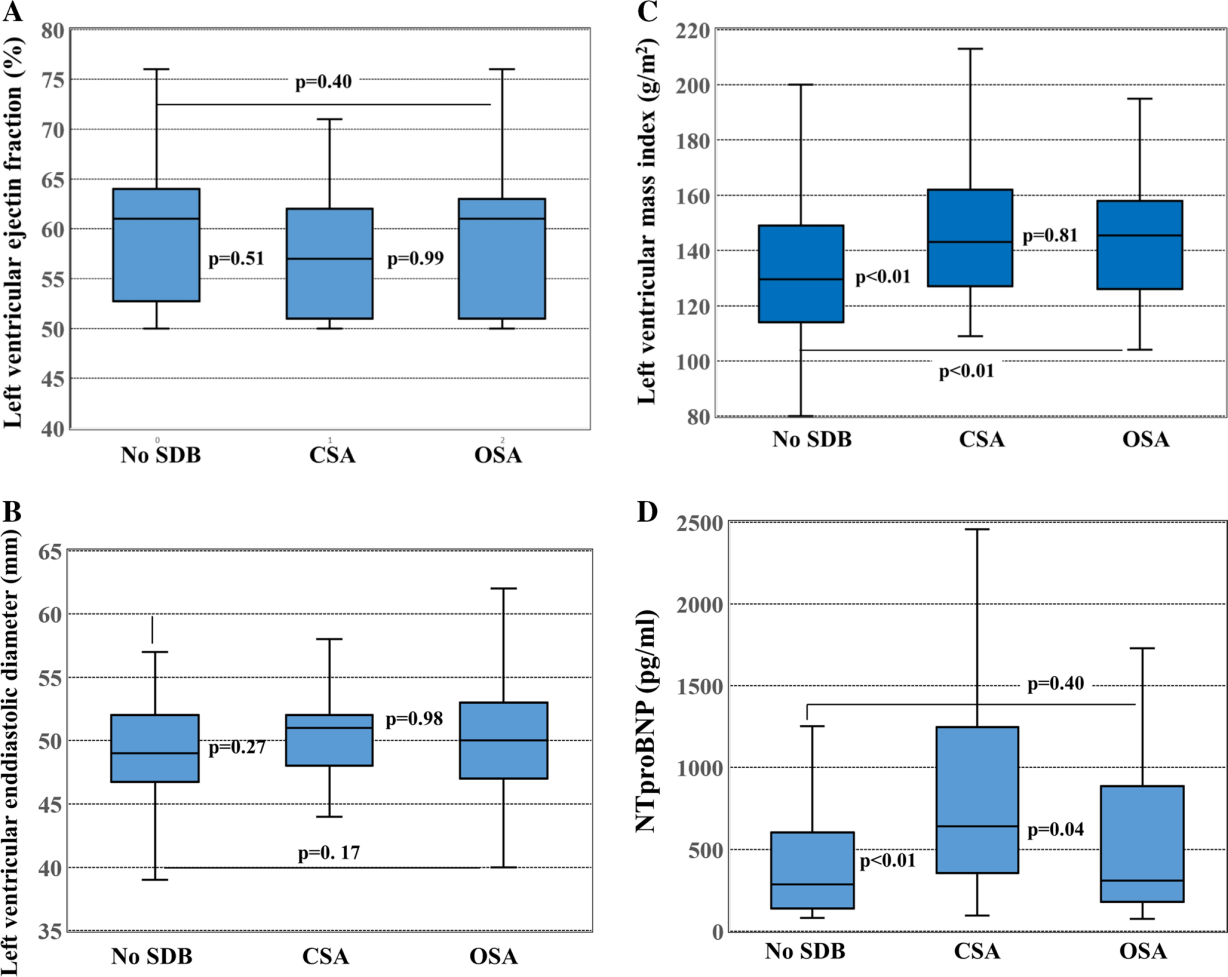
Box plots comparing patients without sleep-disordered breathing (SDB) to patients with central sleep apnea (CSA) and to patients with obstructive sleep apnea (OSA). (**A**) left ventricular ejection fraction was similar in all 3 groups. (**B**) left ventricular end-diastolic diameter was similar in all 3 groups. (**C**) left ventricular mass index was higher in OSA and in CSA compared to no SDB. (**D**) NTproBNP values were significantly higher in CSA compared to OSA and no SDB

Clinical characteristics, comorbidities and echocardiographic findings of 33 patients with predominantly CSA versus 64 patients with predominantly OSA are summarized in Table 2. Mean AHI was similar in patients with CSA compared to OSA (Fig. 2A). Mean ESS score and mean BMI were significantly higher in patients with OSA compared to patients with CSA and to patients without SDB (Fig. 2B and C). Patients with CSA were significantly older (Fig. 2D) and had higher NT-proBNP levels and a higher NYHA heart failure class compared to patients with OSA and to patients without SDB. In addition, patients with CSA more often had aortic valve stenosis, higher HFA-PEFF scores, and were treated more frequently with loop diuretics compared to patients with OSA. Echocardiographic parameters of systolic function including left ventricular ejection fraction and left ventricular size were comparable between patients with CSA, patients with OSA and patients without SDB (Figs. 3A and B). Echocardiographic parameters of diastolic function including E/E’ ratio and left ventricular mass index were significantly higher in patients with CSA and with OSA compared to patients without SDB (Fig. 3C).

Multivariate analysis revealed a significant association between OSA and male gender, BMI and LV mass index, whereas CSA was associated with a significantly higher HFA-PEFF score in addition to male gender (Table 3).

**Table 3:** Predictors of sleep disordered breathing by multivariate analysis

Predictors of OSA	OR (95% Cl)	*P* value
Male gender	1.18 (1.03–1.35)	0.02
BMI (per 5 kg/m^2^ increase)	1.07 (1.02–1.13)	0.02
LV mass index (per 10 g/m2 increase)	1.03 (1.01–1.06)	0.01
**Predictors of CSA**	**OR (95% Cl)**	**P value**
Male gender	1.12 (1.01–1.25)	0.03
HFA-PEFF Score (per 1 increase)	1.10 (1.03–1.17)	< 0.01

BMI, body mass index; OR, odds ratio; CI, confidence interval; LV left ventricular

## Discussion

This study investigated the prevalence and characteristics of SDB in a monocenter cohort with more than 200 patients with HFpEF. As a result, SDB using an AHI cut-off ≥ 15/h was a frequent comorbidity in patients with HFpEF with a higher prevalence of OSA compared to CSA. Importantly, patients with CSA differed from patients with OSA significantly with regard to clinical characteristics, comorbidities, NT-proBNP levels and echocardiographic findings. Patients with CSA had more advanced heart failure as indicated by a higher HFA-PEFF score, a higher NYHA heart failure class and higher NT-proBNP levels, whereas patients with OSA had a higher BMI and a higher ESS score indicating increased daytime sleepiness.

### Previous studies investigating SDB in HFpEF

The results of 11 previous studies reporting the prevalence of SDB in patients with HFpEF are summarized in Table 4. The reported prevalence of SDB in these studies varied considerably between 36% in the large German SchlaHF-XT multicentre study reported by Arzt et al.[11] and 83% in the study by Kishan et al.[9]. This wide range of SDB prevalence in HFpEF patients in previous studies may in part be explained by several important differences between these studies. Only three previous studies [2, 6, 8] enrolling small numbers of HFpEF patients used gold standard polysomnography, whereas all other studies [3–5, 7, 9–12] including our present study used polygraphic devices, which can underestimate the extent of SDB and particularly CSA. Furthermore, 5 previous studies [3, 7, 9–11] excluded patients with a history of SDB from study enrollment, whereas the remaining 6 studies [2, 4–6, 8, 10] did not exclude patients with known SDB. Finally, previous studies used different methods to diagnose HFpEF as well as various scoring rules for OSA and CSA. Importantly, previous studies used different AHI cut-offs as summarized in Table 4. When using AHI cut-offs defining respiratory events, one has to keep in mind that AHI has shortcomings since AHI primarily captures the number of apneas and hypopneas while ignoring the lengths of apneas and other important forms of SDB, such as flow limitation or respiratory effort-related arousals or disruptions in sleep without meeting the thresholds for apneas or hypopneas.

**Table 4:** Previous studies investigating the prevalence of SDB in patients with HFpEF

Author	Year	Patients, *n*	Method	Definition	SDB, %	OSA, %	CSA,%
Chan et al.[2]	1997	20	PSG	AHI ≥ 10/h	55	35	20
Bitter et al.[3]	2009	244	PG	AHI ≥ 5/h	69	40	29
Herrscher et al.[4]	2011	44	PG	AHI ≥ 5/h	80	62	18
Sekizuka et al.[5]	2013	19	PG	AHI ≥ 15/h	37	11	26
Arikawa et al.[6]	2016	58	PSG	AHI ≥ 5/h	67	67	0
Borrelli et al.[7]	2019	175	PG	AHI ≥ 5/h (AHI ≥ 15/h)	79 (44)	41 (21)	38 (23)
Gupta et al.[8]	2020	25	PSG	AHI ≥ 10/h	64	52	12
Kishan et al.[9]	2021	46	PG	AHI ≥ 5/h	83	61	22
Huang et al.[10]	2022	242	PG	AHI ≥ 15/h	44	29	15
Arzt et al.[11]	2022	2032	PG	AHI ≥ 15/h	36	29	7
Wang et al.[12]	2023	173	PG	AHI ≥ 5/h	62	49	13
Present study	2025	233	PG	AHI ≥ 15/h (AHI ≥ 5/h)	42 (74)	27 (55)	14 (19)

AHI, apnea-hypopnea index; CSA, central sleep apnea; OSA, obstructive sleep apnea;

PG, polygraphy; PSG, full polysomnography; SDB, sleep disordered breathing

Using the same AHI cut-off ≥ 15/h to define moderate to severe SDB as Arzt and coworkers [11], we found a 42% prevalence of SDB in HFpEF, which is only slightly higher than the prevalence of 36% reported by Arzt and colleagues [11]. When using an AHI cut-off ≥ 5/h to define SDB in our study, the prevalence of SDB in our study rises to 74%, which is comparable to the SDB prevalence of 79% reported by Borrelli et al.[7] and the prevalence of 80% reported by Herrscher et al.[4] who used an AHI cut-off ≥ 5/h to define SDB in HFpEF. If Borrelli et al.[7] had used an AHI cut-off ≥ 15/h to define SDB, the prevalence of SDB decreased to 44% (Table 4).

The prevalence of 14% for CSA in HFpEF patients in our study does not confirm the high prevalence of CSA in HFpEF of 29% reported by Bitter et al.[3] and 38% reported by Borrelli et al.[7] or the low prevalence of CSA in HFpEF of 7% in the SchlaHF-XT study by Arzt et al.[11]. The discrepancies between these studies may in part be explained by differences in patient populations as well as various scoring rules for OSA and CSA. The prevalence of 14% for CSA in our study is comparable to the results reported by Herrscher et al.[4], Gupta et al.[8], Huang et al.[10] and Wang et al.[12], who reported a CSA prevalence ranging from 12% to 18% (Table 4).

In contrast to our present study, Arzt et al.[11] and Wang et al.[12] also investigated the prevalence of SDB in patients with heart failure with reduced left ventricular ejection fraction (HFrEF) in addition to HFpEF. Both investigators found a significantly higher prevalence of CSA in HFrEF compared to HFpEF suggesting an inverse relationship between CSA prevalence and LV ejection fraction. This finding is consistent with the results of a previous study at our institution in patients with HFrEF [23], in which we found a 43% prevalence of CSA using an AHI cut-off ≥ 15/h in patients with HFrEF versus 14% in patients with HFpEF in our present study.

### Predictors of SDB in patients with HFpEF

By univariate comparison of patients with and without SDB, several previous studies reported a significant association between SDB and male gender [7, 9, 11], NT-proBNP or BNP levels [6, 7], body mass index [7, 9, 11, 12], NYHA heart failure class [11], older age [11], and various parameters for the degree of diastolic dysfunction [2, 3, 8] in patients with HFpEF. Comparability of these findings, however, is limited by several factors. Most previous studies did not evaluate potential important predictors of SDB simultaneously and only 4 previous studies [7, 9–11] used multivariate analyses. In addition, several previous studies did not report NT-proBNP or BNP values [2, 9, 11] as recommended by current heart failure guidelines of the European Society of Cardiology [13, 14], while other studies did not report any parameters of echocardiographic diastolic dysfunction for patients with SDB compared to patients without SDB2, [4, 8–10].

Similar to our study, Bitter et al.[3] found significantly higher NT-proBNP values in patients with CSA but not in patients with OSA compared to patients without SDB suggesting that patients with HFpEF and CSA have more severe heart failure than patients with HFpEF and OSA or patients with HFpEF without SDB.

Similarly, Borrelli et al.[7] described significantly higher NT-proBNP levels and age as predictors of nocturnal CSA, whereas BMI and arterial hypertension predicted OSA during nighttime. Using multivariate analysis, Borrelli et al.[7] found no predictor for CSA at nighttime, whereas we could demonstrate that the HFA-PEFF score [21], which is exclusively based on natriuretic peptide levels and echocardiographic measurements of diastolic dysfunction and age are predictors of CSA in patients with HFpEF by multivariate analysis. In contrast to patients with CSA, multivariate analysis revealed a significant association between OSA and male gender, BMI and LV mass index but not NT-proBNP levels in our study.

Despite several design similarities between our study and previous studies [2–12], our study has important strengths compared to previous studies. First, the most important comorbidities of HFpEF, NT-proBNP levels, renal function as well as comprehensive echocardiographic evaluation of diastolic dysfunction [15] were determined prospectively in all study patients as recommended by current heart failure guidelines of the European Society of Cardiology [13, 14]. Secondly, we enrolled 233 patients with HFpEF, whereas only 3 of 12 previous studies [3, 10, 11] listed in Table 4 enrolled more than 200 patients with the largest patient cohort being reported by Arzt et al.[11] in the SchlaHF-XT study. Our study is, however, to our best knowledge, the first study which calculated both H_2_FPEF and HFA-PEFF scores in all patients as recommended by current heart failure guidelines of the European Society of Cardiology [13, 14, 21].

### Study limitations

Since many patients with HFpEF were enrolled during their first visit at our cardiology outpatient department, only approximately half of the patients were treated with sGLT2-inhibitors, which have been shown to improve prognosis in HFpEF and have received a class I indication for HFpEF therapy in current heart failure guidelines [3, 14]. To address this limitation, adjustment for all patients’ medications listed in Table 1 as potential confounding factors was performed during multivariate analysis. Another limitation is the use of cardiorespiratory polygraphy rather than gold standard polysomnography to detect SDB. Polygraphy can underestimate CSA, especially hypopnea-related central events. Furthermore, CSA in our study was defined based on predominance of central apneas, rather than the proportion of all central respiratory events including hypopneas. Finally, the number of patients with CSA in our study was modest, which limits statistical power for multivariate analysis and residual confounding remains possible, particularly from unmeasured factors.

## Conclusions

Moderate to severe SDB is a frequent comorbidity in patients with HFpEF. Predominantly OSA is more frequent than CSA with significant clinical differences between patients with predominantly OSA and CSA. Whereas OSA is associated with a higher BMI in addition to male gender, CSA is associated with more advanced heart failure as indicated by a higher HFA-PEFF score, a higher NYHA heart failure class and higher NT-proBNP levels. Potential therapeutic and prognostic implications resulting from the observed differences between HFpEF with CSA and HFpEF with OSA warrant investigation in well-designed future studies in large patient cohorts with HFpEF.

## Data Availability

The datasets generated during and/or analysed during the current study are available from the corresponding author on reasonable request.
